# Non-canonical NFκB mutations reinforce pro-survival TNF response in multiple myeloma through an autoregulatory RelB:p50 NFκB pathway

**DOI:** 10.1038/onc.2016.309

**Published:** 2016-09-19

**Authors:** P Roy, T Mukherjee, B Chatterjee, B Vijayaragavan, B Banoth, S Basak

**Affiliations:** 1 Systems Immunology Laboratory, National Institute of Immunology, New Delhi, India

## Abstract

Environmental drug resistance constitutes a serious impediment for therapeutic intervention in multiple myeloma. Tumor-promoting cytokines, such as tumor necrosis factor (TNF), induce nuclear factor-κB (NFκB)- driven expression of pro-survival factors, which confer resistance in myeloma cells to apoptotic insults from TNF-related apoptosis-inducing ligand (TRAIL) and other chemotherapeutic drugs. It is thought that RelA:p50 dimer, activated from IκBα-inhibited complex in response to TNF-induced canonical NFκB signal, mediates the pro-survival NFκB function in cancerous cells. Myeloma cells additionally acquire gain-of-function mutations in the non-canonical NFκB module, which induces partial proteolysis of p100 into p52 to promote RelB:p52/NFκB activation from p100-inhibited complex during immune cell differentiation. However, role of non-canonical NFκB signaling in the drug resistance in multiple myeloma remains unclear. Here we report that myeloma-associated non-canonical aberrations reinforce pro-survival TNF signaling in producing a protracted TRAIL-refractory state. These mutations did not act through a typical p52 NFκB complex, but completely degraded p100 to reposition RelB under IκBα control, whose degradation during TNF signaling induced an early RelB:p50 containing NFκB activity. More so, autoregulatory RelB synthesis prolonged this TNF-induced RelB:p50 activity in myeloma cells harboring non-canonical mutations. Intriguingly, TNF-activated RelB:p50 dimer was both necessary and sufficient, and RelA was not required, for NFκB-dependent pro-survival gene expressions and suppression of apoptosis. Indeed, high RelB mRNA expressions in myeloma patients correlated with the augmented level of pro-survival factors and resistance to therapeutic intervention. In sum, we provide evidence that cancer-associated mutations perpetuate TNF-induced pro-survival NFκB activity through autoregulatory RelB control and thereby exacerbate environmental drug resistance in multiple myeloma.

## Introduction

Multiple myeloma, an incurable plasma cell malignancy, accounts for ~13% of all hematological cancers.^1^ Disease progression involves clonal expansion of transformed plasma cells into tumors in the bone marrow. TNF-related apoptosis-inducing ligand (TRAIL) and other chemotherapeutic drugs induce Caspase-8 dependent apoptosis in various cancerous cells with negligible toxicity for healthy cells.^2^ Preclinical studies accordingly advocated for TRAIL-based interventions in multiple myeloma.^3, 4, 5^ However, subsequent clinical trials revealed a rather moderate benefit with only a small subset of patients responding satisfactorily to TRAIL.^2, 6^ Indeed, certain patient-derived myeloma cells were found to be resistant to drug-induced apoptosis owing to increased level of cFLIP and other pro-survival factors, which counteract caspase activation.^4^ As drug resistance constitutes a serious impediment for myeloma therapy, it is important to understand fully the underlying molecular mechanisms.

In myeloma cells, tumor necrosis factor (TNF) induces expression of the pro-survival factors, which are known to confer resistance to apoptotic insults, including TRAIL.^7, 8, 9^ Bone marrow stromal cells provide paracrine TNF signals in myeloma.^10^ Serum level of TNF was correlated with disease severity in multiple myeloma,^11, 12^ and provided for a predictive indicator of high symptom burden for patients undergoing maintenance therapy.^13^ Clinical trials also demonstrated that thalidomide analogs, which inhibit TNF production in the tumor microenvironment, enhance overall response in TRAIL-based therapy.^14, 15^ These studies implicated TNF in environmental-mediated drug resistance in multiple myeloma.

At the molecular level, TNF engages NEMO (nuclear factor-κB (NFκB_ essential modulator)-IKK2 (IκB kinase subunit 2, also known as IKKβ) kinase complex, which promotes phosphorylation and degradation of inhibitory IκBα (inhibitor of NF-κBα), thereby, liberating RelA:p50 dimer into the nucleus via the canonical NFκB pathway.^16^ In a negative feedback loop, RelA:p50 transcriptionally induces synthesis of IκBα, which ensures post-induction attenuation of RelA:p50/NFκB activity. TNF stimulates transcription of pro-survival factors from their cognate κB-driven promoters. It is commonly believed that RelA:p50 mediates this pro-survival NFκB function in myeloma cells.^7^ Importantly, IKK inhibitors were shown to sensitize myeloma cells to apoptotic death.^17, 18^

In addition, a distinct non-canonical NFκB pathway is activated by cell-differentiating cues in physiological settings.^19^ In resting cells, cIAP1/cIAP2 and TRAF3-dependent ubiquitination promotes degradation of NIK (NFκB inducing kinase). Induction of the non-canonical pathway rescues NIK from constitutive degradation resulting in activation of IKK1 (IκB kinase subunit 1, also known as IKKα). IKK1 phosphorylates *Nfkb2-*encoded precursor p100, which retains RelB in the cytoplasmic complex in unstimulated cells. Subsequent partial proteolysis or processing of p100 removes the C-terminal inhibitory domain to generate mature p52 subunit, thereby liberating RelB:p52/NFκB activity into the nucleus. Myeloma cells acquire gain-of-function mutations in this non-canonical NFκB pathway with separate studies reporting ~10–15% multiple myeloma patients harbor such genetic aberrations.^18, 20, 21^ Myeloma-associated mutations either inactivate negative regulators of NIK, such as cIAP1/cIAP2 and TRAF3, or augment expression of upstream inducers of the pathway, including CD40, LTβR as well as NIK itself.^18, 22, 23^ Moreover, mutations in *Nfkb2* that produce dysfunctional p100 lacking the NFκB inhibitory domain has been reported.^24^ It was proposed that NIK activates the canonical IKK2-RelA pathway in a subset of human myeloma cell lines (HMCLs) to promote cell survival.^18, 22, 25^ However, a role of NIK-induced non-canonical RelB/NFκB activity in imparting drug resistance in multiple myeloma has not been explored.

The RelB/NFκB-mediated gene expressions is thought to have limited physiological functions in immune cell differentiation.^19^ Interestingly, a low level of NIK-independent, constitutive RelB:p52/NFκB activity has been identified in myeloma cells.^26^ Surprisingly, constitutive RelB:p52 activity was shown both to promote and suppress NFκB-dependent gene expressions in HMCLs and patient-derived myeloma cells.^26, 27^ As NFκB target genes encode important pro-survival factors, we asked if mutational activation of the non-canonical RelB/NFκB pathway modulates TNF response, and thereby modifies resistance of myeloma cells to apoptotic insults.

Here, we demonstrate that non-canonical NFκB mutations collaborate with TNF signaling in producing a protracted TRAIL-refractory state in myeloma cells. In an interdisciplinary approach, we identify that depletion of p100, owing aberrations in the non-canonical module, triggers an autoregulatory RelB pathway, which perpetuates an alternate RelB:p50 NF-κB activity in response to TNF. Intriguingly, pro-survival TNF response is attributed to RelB:p50, and not RelA:p50, NFκB activity in myeloma cells harboring non-canonical mutations. Finally, our database analyses indicate a possible role of autoregulatory RelB/NFκB pathway in resistance of myeloma patients from therapeutic intervention. In sum, we provide evidence that cancer-associated mutations perpetuate TNF-induced pro-survival NFκB response through autoregulatory RelB control and thereby exacerbate environmental drug resistance in multiple myeloma.

## Results

### Non-canonical NFκB mutations collaborate with TNF in exacerbating resistance of myeloma cells to apoptotic insults

**Figure 1:**
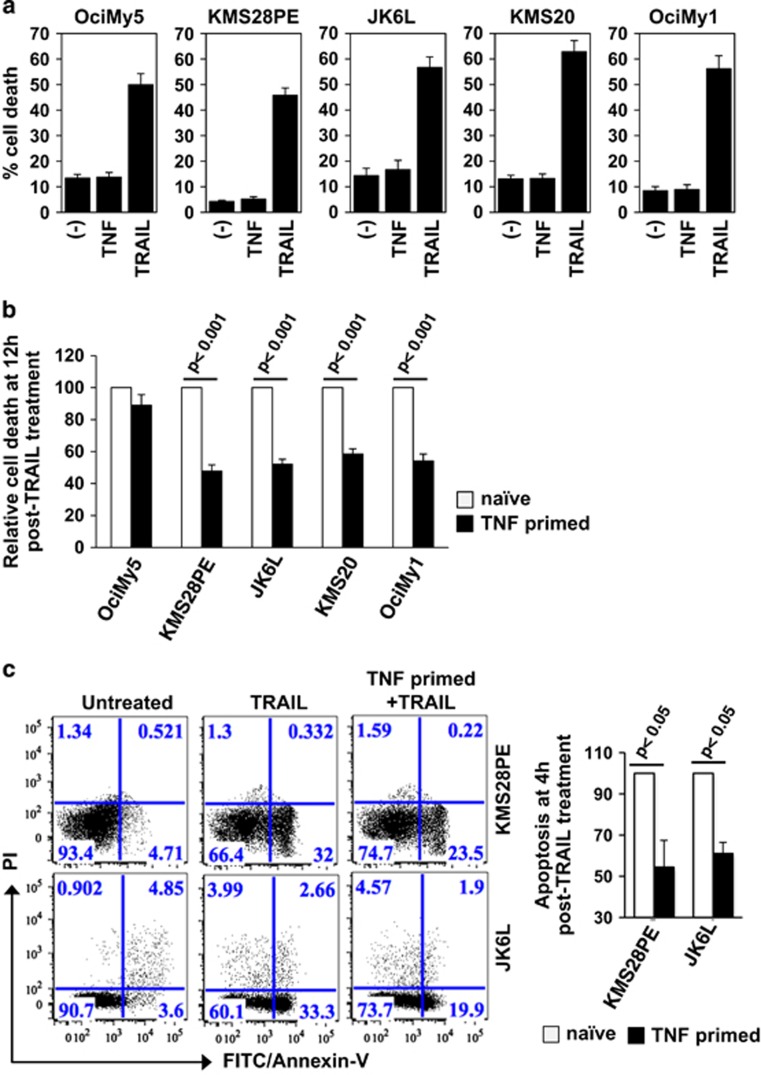
Non-canonical NFκB mutations exacerbate protracted TRAIL resistance in TNF-primed myeloma cells. (**a**) TNF- or TRAIL-mediated cell death was assessed at 12 h post-treatment in indicated human myeloma cell lines (HMCLs) using trypan blue dye exclusion assay. Basal cell death in the untreated cell has also been presented. The results reveal mean of five biological replicates±s.e.m. (**b**) Indicated HMCLs were primed with 10 ng/ml of TNF for 8 h and then treated with TRAIL. TRAIL-mediated cell death was assessed at 12 h post-TRAIL treatment and presented relative to the cell death induced in respective naïve cells treated with TRAIL alone subsequent to subtracting for basal cell death. The plot represents average from six independent biological replicates±s.e.m. (**c**) Naïve or TNF-primed KMS28PE and JK6L cells were treated with TRAIL for 4 h, stained and subjected to fluorescence-activated cell sorting. In a dot plot, population of cells undergoing apoptosis (Annexin-V^+^-PI^−^) was determined. Untreated cells were used as control. Right, a bar diagram revealing fraction of apoptotic cells in naïve and TNF-primed sets after TRAIL treatment. The data represent average of four independent biological replicates corrected for basal apoptosis in respective sets.

Given prevalence of mutations in the non-canonical NFκB module in multiple myeloma, we examined if these mutations alter resistance of cancerous cells to apoptotic TRAIL. We analyzed HMCLs with mutations in the non-canonical pathway; KMS28PE as well as KMS20 harbor genetic aberration in locus encoding cIAPs, OciMy1 bears homozygous deletion of *Traf3*, and JK6L contains an inactivating mutation in *Nfkb2*/p100.^18, 22, 28^ As a control, we used OciMy5 cells that lack non-canonical mutations but bear an amplification of *Nfkb1*. However, cell treatment using 100 ng/ml of TRAIL led to similar ~45-55% cell death at 12 h post-treatment in these HMCLs irrespective of mutations in non-canonical module (Figure 1a and Supplementary Figure S1a). TNF treatment alone had no discernible effect on cell viability (Figure 1a). It has been suggested that TNF instill a refractory state in myeloma cells present in the bone marrow that then become resistant to TRAIL and other chemotherapeutic agents.^8, 9^ To capture this pathological role of TNF, we primed myeloma cells with TNF and subsequently exposed them to TRAIL at 8 h post-TNF treatment. Interestingly, TNF priming resulted in ~50% protection from TRAIL-mediated cell death specifically in HMCLs harboring non-canonical mutations, such as KMS28PE, JK6L, KMS20 and OciMy1, with only marginal effect in control OciMy5 cells (Figure 1b). Our fluorescence-activated cell sorting analyses revealed early apoptotic death in 32% KMS28PE and 33.3% JK6L cells within 4 h of TRAIL treatment (Figure 1c and Supplementary Figure S1b) and also consistently demonstrated that TNF priming led to more than 40% reduction in TRAIL-induced apoptotic cell death in KMS28PE as well as JK6L cells (Figure 1c). These results indicated that activating mutations in the non-canonical NFκB module might not act independently, but collaborate with TNF in exacerbating resistance to chemotherapeutic drugs in multiple myeloma.

### Perpetuating RelB:p50/NFκB activity confers TRAIL resistance in TNF-primed HMCLs harboring non-canonical mutations

**Figure 2:**
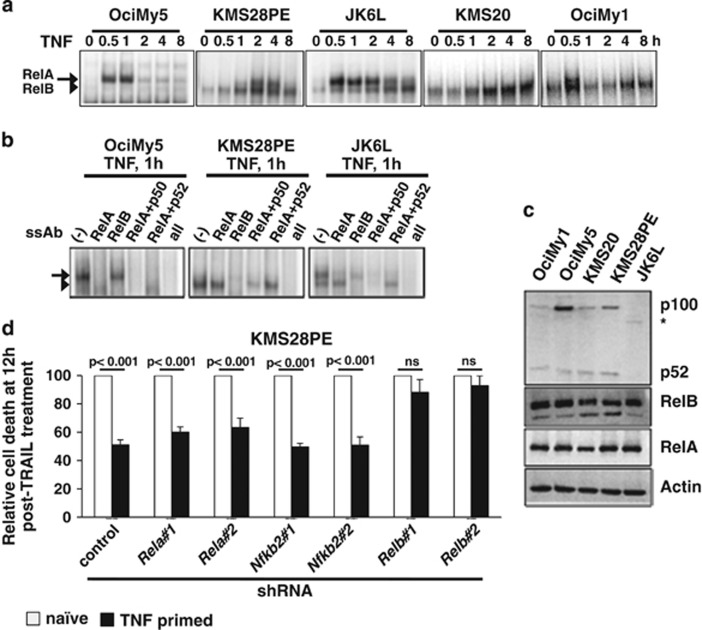
Non-canonical mutations provoke prolonged RelB:p50/NFκB response to TNF in generating TRAIL resistance in HMCLs. (**a**) Nuclear NF-κB activity induced in a TNF time course in OciMy5, KMS28PE, JK6L, KMS20 and OciMy1 was resolved in electrophoretic mobility shift assay (EMSA) using a κB site containing DNA probe. Arrow and arrowhead represent RelA and RelB containing NFκB DNA-binding dimers, respectively, as determined in (**b**). (**b**) Composition of TNF-induced nuclear NFκB DNA-binding activity was examined using supershift assay in OciMy5 as well as in KMS28PE and JK6L cells that represented HMCLs harboring non-canonical mutations. Ablation of respective DNA-binding complexes using specific antibodies indicated that the slower migrating complex on EMSA gel is composed of RelA and the faster migrating complex is composed of RelB. Combining αp50 or αp52 antibody with αRelA antibody, composition of the residual RelB NFκB DNA-binding activity was determined. (**c**) Immunoblot charting cellular levels of p52/p100, RelB and RelA in a panel of HMCLs. * indicates a truncated p100 protein expressed in JK6L. (**d**) Effect of shRNA-mediated knockdown of RelA, NFκB2 or RelB expression in KMS28PE on TNF priming was captured in trypan blue dye exclusion based cell-death assay. Two different shRNAs have been used for each gene. The results reflect average of five independent experiments±s.e.m.

Because TNF-induced pro-survival mechanisms are thought to involve the canonical NFκB pathway, we tested whether these non-canonical mutations strengthen TNF-activated RelA/NFκB response. Our electrophoretic mobility shift assay (EMSA) and supershift analyses revealed that TNF induced comparable nuclear RelA NFκB DNA-binding activity, which was similarly attenuated by 8 h in OciMy5, KMS28PE, JK6L and OciMy1 cell lines regardless of non-canonical mutations (Figures 2a and b). TNF signal led to very weak, if any, RelA NFκB activation in KMS20 cells. Curiously, TNF activated an additional RelB:p50 containing NFκB dimer in HMCLs with non-canonical mutations, but not in control OciMy5 cell line. Unlike diminishing RelA:p50 activity, RelB:p50 dimer progressively accumulated in the nucleus of these cells producing robust κB DNA-binding activity at 8 h post-stimulation (Figures 2a and b). Induction of canonical NFκB signaling using PMA/Ionomycin also induced prolonged RelB:p50 response in JK6L cells (Supplementary Figure S2a). Prior studies have suggested that p100, instead of being partially proteolysed into p52, undergoes complete degradation in myeloma-derived cells in the presence of chronic non-canonical signal.^29, 30^ As RelB:p52 constituted only a minor NFκB DNA-binding activity in KMS28PE and JK6L cells (Figure 2b), we examined whether activating mutations in the non-canonical module led to degradation of p100. Our immunoblot analyses revealed a severe reduction in p100 levels in KMS28PE, KMS20 and OciMy1 cells, as opposed to control OciMy5 cell line (Figure 2c). Consistent to earlier observation on p100 degradation,^29^ we were unable to detect p52 accumulation concomitant to p100 depletion in HMCLs possessing non-canonical mutations. Likewise, JK6L expressed an unstable form of truncated p100 only at a reduced level without p52 accumulation. However, NFκB2 mRNA levels were comparable in these HMCLs (Supplementary Figure S2b). We also observed significantly a reduced level of RelA protein in KMS20 cells (Figure 2c), which likely have contributed to the diminished RelA/NFκB activity in these cells.

Next, we investigated role of individual NFκB dimers in HMCLs bearing distorted non-canonical module. We found that shRNA-mediated knockdown of RelA expression does not impact TRAIL resistance of TNF-primed KMS28PE cells in our cell-death assay (Figure 2d and Supplementary Figure S2c). Intact TRAIL resistance of KMS28PE cells subjected to knockdown of NFκB2 mRNA suggested that p52 is dispensable for the protective effect of TNF (Figure 2d and Supplementary Figure S2d). Remarkably, shRNA depletion identified that RelB is critical for the resilience of TNF-primed KMS28PE cells to subsequent TRAIL treatment (Figure 2d and Supplementary Figure S2e). Our data indicated that prolonged RelB/NFκB activity induced by TNF is responsible for the protracted drug-refractory state of HMCLs harboring non-canonical aberrations, and that this resistance is independent of RelA. These studies also raised a mechanistic conundrum as activating mutations in the non-canonical NFκB module do not appear to act through RelB:p52; rather they promote perpetuating RelB:p50/NFκB response to canonical TNF signal.

### A lack of p100 promotes RelB:p50/NFκB activation upon TNF stimulation

**Figure 3:**
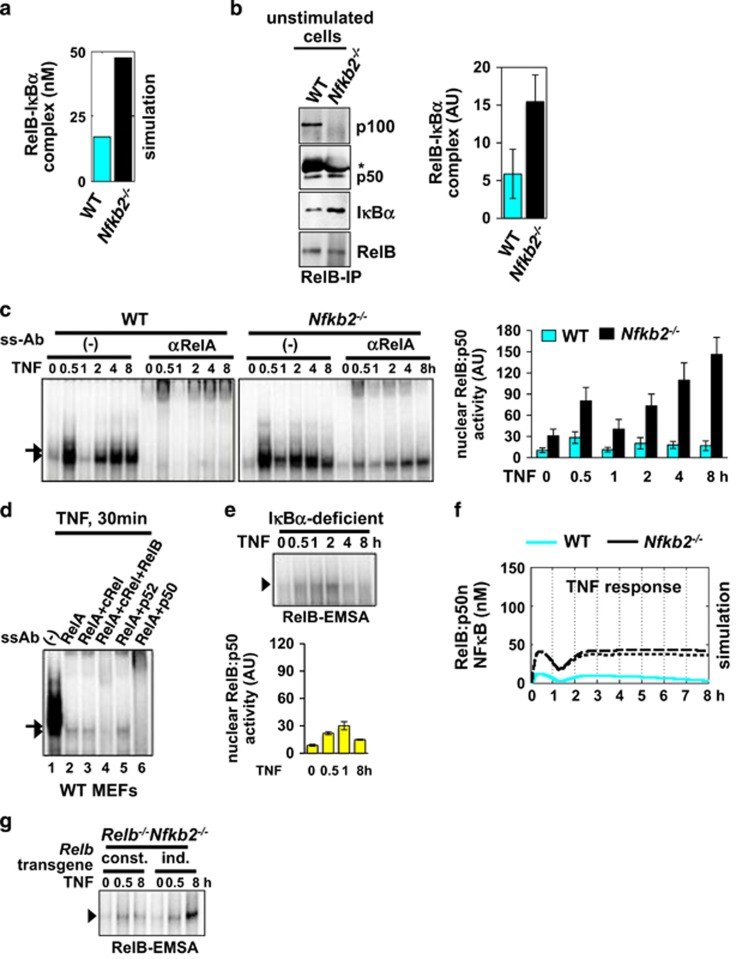
TNF induces prolonged NFκB activity composed of RelB:p50 dimer in the absence of p100. (**a**) Computational simulation predicting abundance of latent RelB complexes bound to IκBα in WT and in *Nfkb2*^−/−^ cell systems. (**b**) Immunoblot of RelB co-immunoprecipitates, normalized for RelB content, derived from WT or *Nfkb2*^−/−^ MEFs extracts. * denotes IgG heavy chain. Right, quantification of the intensity of the band corresponding to coimmunoprecipitated IκBα. The data represent three independent experiments. (**c**) Left, EMSA demonstrating nuclear NFκB activity in a time course upon 1 ng/ml of TNF stimulation of WT or *Nfkb2*^−/−^ MEFs. Supershifting RelA complexes in EMSA, TNF-induced RelB dimer was revealed. Right, signal corresponding to RelB/NFκB activity was quantified and expressed as mean of four biological replicates±s.e.m. (**d**) Composition of NFκB activity induced in WT MEFs upon 30 min of TNF stimulation was examined by supershift assay. Ablating RelA DNA-binding activity, residual RelB:DNA complex was revealed. Combining αp50 or αp52 antibody with αRelA antibody, composition of the RelB DNA-binding activity was determined. (**e**) Supershifting RelA, TNF-induced nuclear activation of RelB/NFκB dimer in IκBα-deficient MEFs was revealed in RelB-EMSA. Bottom, quantified RelB/NFκB signal was presented. (**f**) Computational simulation revealing TNF activation of the RelB:p50 dimer in WT (cyan) and *Nfkb2*^−/−^ (black) MEFs. Dotted and dashed black lines represent RelB:p50 induction in NFκB2-null system without or with RelA induced *Relb* synthesis control, respectively. (**g**) RelB-EMSA showing early (0.5 h) and late (8 h) RelB:p50 induction by TNF in *Relb*^−/−^*Nfkb2*^−/−^ MEFs stably expressing RelB from either a constitutive (const.) or an NF-κB inducible (ind.) promoter. The data represents two independent experiments.

How do aberrations in the non-canonical module instruct altered NFκB dimer usage during canonical signaling? In resting cells, RelB is thought to be sequestered in RelB:p100 complex, which is insensitive to canonical signals. In specialized cell types, however, RelB:p50 dimer was shown to bind to IκBα and translocate into the nucleus upon IκBα degradation.^31^ To further understand RelB/NFκB control in inflammatory settings, we rewired p100- and IκBα-mediated regulations in a mathematical model, termed the NFκB Systems Model *v*2.0 (see Supplementary Appendix for a detailed description). Our computational simulations suggested that a small fraction of RelB is sequestered by IκBα in unstimulated WT cells, and that abundance of RelB–IκBα complex is increased by threefold in the p100-deficient system (Figure 3a). We tested these predictions using genetically defined mouse embryonic fibroblasts (MEFs). Our immunoprecipitation analyses demonstrated that RelB associates with p100 as well as p50, and also binds to IκBα in WT cells. Moreover, abundance of RelB–IκBα complex was augmented in *Nfkb2*^−/−^ MEFs (Figure 3b). RelB also bound to IκBɛ in the absence of p100 (Supplementary Figure S3a). We concluded that the absence of p100 facilitates complex formation between RelB and classical IκBs, which respond to canonical NF-κB signal.

Consistently, elevated level of latent RelB–IκB complex promoted induction of nuclear RelB:p50/NFκB activity within 30 min of TNF stimulation in *Nfkb2*^−/−^ MEFs with only minor RelB:p50 induction in WT cells (Figures 3c and d and Supplementary Figure S3b). Although this early RelB:p50 activity was attenuated by 1 h, TNF additionally stimulated a late RelB:p50 NFκB DNA-binding activity, which gradually accumulated in the nucleus between 2  and 8 h in *Nfkb2*^−/−^ MEFs (Figure 3c). Defective attenuation of early RelB:p50 activity at 1 h in IκBα-deficient MEFs (Figure 3e) suggested that IκBα negative feedback regulates the duration of early RelB:p50 response, analogous to RelA:p50 control. Absence of IκBα feedback, however, did not promote RelB:p50 activity at 8 h in IκBα-deficient MEFs (Figure 3e) indicating that additional mechanisms potentiate this late activity.

Although our computational simulation faithfully reproduced early RelB:p50 induction, it failed to capture progressive nuclear accumulation of late-acting RelB:p50 dimer observed in TNF-stimulated *Nfkb2*^−/−^ MEFs (Figure 3f). It was shown that RelA:p50 induces the expression of RelB mRNA from the κB-driven promoter (Supplementary Figures S3c and S4a).^32^ We hypothesized that RelA-driven RelB synthesis promotes this late RelB:p50 activity. TNF stimulation of *Relb*^−/−^*Nfkb2*^−/−^ MEFs expressing RelB from either a constitutive or a κB-driven retroviral promoter (Supplementary Figure S3d) confirmed that κB-dependent expression of RelB is necessary for late, but not early, RelB:p50 induction in the absence of p100 (Figure 3g). Our immunoprecipitation analyses indicated that p100 sequesters RelB to curtail this late-acting RelB:p50 response in WT cells (Supplementary Figure S3e).^33^ Even so, implementation of RelA-driven RelB synthesis in our mathematical model was insufficient for recapitulating late RelB:p50 activity in *Nfkb2*^−/−^ cells (Figure 3f). Our analyses revealed that a lack of p100 not only provokes early RelB:p50 activity, but also unleashed a robust late-acting RelB:p50 response to TNF. This persistent RelB:p50 response in *Nfkb2*^−/−^ cells mirrored the prolonged RelB:p50 activity observed in HMCLs devoid of p100. However, our biochemical and computational studies suggested that well-documented mechanisms, those include IκBα mediated regulations and RelA-induced synthesis control, are inadequate to account for the prolonged RelB:p50/NFκB response during canonical TNF signaling.

### An autoregulatory circuit prolongs RelB:p50/NFκB activity upon TNF stimulation of p100-deficient system

**Figure 4:**
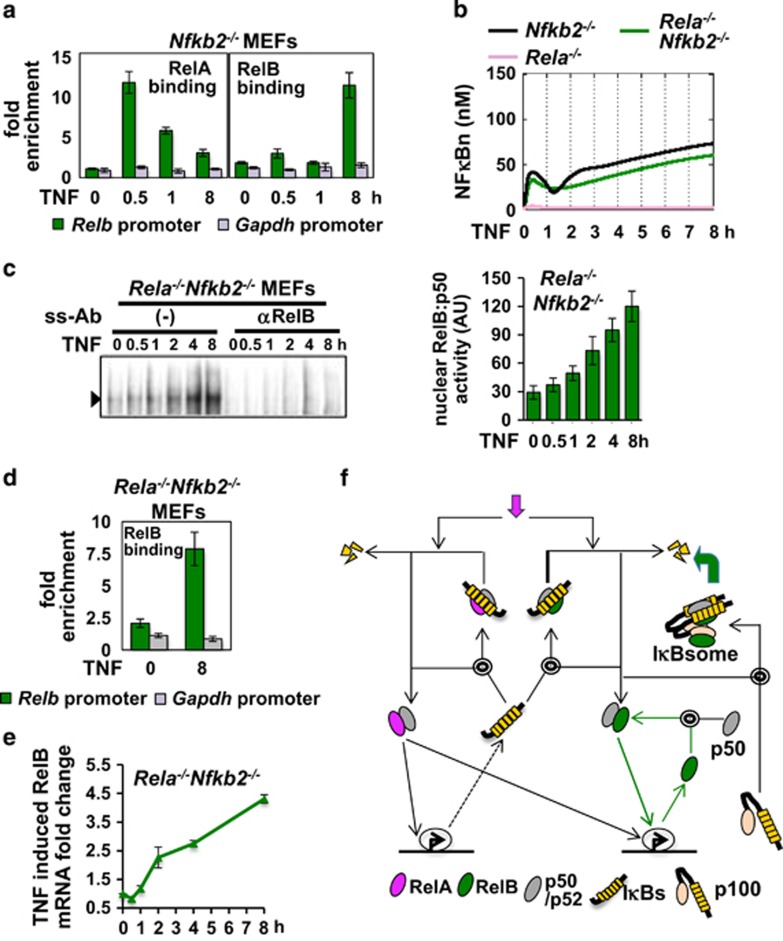
An autoregulatory pathway propagates TNF-induced RelB:p50 activity in p100-deficient cells. (**a**) ChIP analyses, representative of three independent biological replicates, revealing recruitment of RelA (left) and RelB (right) NFκB dimers to *Relb* promoter in a TNF time course in *Nfkb2*^−/−^ MEFs. Fold enrichment of *Relb* promoter DNA in RelA or RelB immunopellet relative to control IgG was determined using quantitative PCR. Binding to *Gapdh* promoter served as a negative control. (**b**) Computational simulation recapitulating progressive nuclear accumulation of the RelB:p50 dimer in response to TNF in *Nfkb2*^−/−^ MEFs upon implementing positive autoregulatory control in the mathematical model. Also, computational studies predicting intact late RelB:p50 response in *Rela*^−/−^*Nfkb2*^−/−^ MEFs. (**c**) Electrophoretic mobility shift assay (EMSA) revealing nuclear translocation of the RelB:p50 dimer in a TNF time course in *Rela*^−/−^*Nfkb2*^−/−^ MEFs. Right, RelB/NFκB signal was quantified and expressed as a mean of four biological replicates±s.e.m. (**d**) ChIP analyses demonstrating TNF-induced recruitment of RelB to *Relb* promoter at 8 h post-stimulation in *Rela*^−/−^*Nfkb2*^−/−^ cells. The data represent three independent experiments. (**e**) Quantitative reverse transcriptase–PCR revealing the relative level of RelB mRNA in a TNF time course in *Rela*^−/−^*Nfkb2*^−/−^ MEFs. The data represent three biological replicates. (**f**) A graphical depiction of the proposed signaling circuitry underlying inflammatory RelB control. RelB either assembles into IκBsome with preferred binding partner p100 or binds to IκBα as the RelB:p50 dimer. TNF signal targets IκB-inhibited complex to transiently activate RelB:p50. In addition to RelA-induced synthesis, a dominant autoregulatory loop, denoted with green line, prolongs RelB:p50 response to TNF in the absence of p100. Magenta and green block arrows signify targeting of IκBα and p100 by canonical and non-canonical signals, respectively.

Although κB sites in the *Relb* promoter were necessary, RelA-driven synthesis of RelB was not sufficient for prolonged RelB:p50/NFκB response to TNF in p100-depleted cell systems (Figures 3f and g). We examined if the RelB:p50 dimer *per se* mediates RelB synthesis in perpetuating RelB:p50/NFκB activity. Our *in vitro* DNA-binding analyses revealed that RelA:p50 and RelB:p50 both bind to the conserved κB sites present in *Relb* promoter with comparable efficiencies (Supplementary Figures S4b and c). Using ChIP assay, we could demonstrate that RelA is recruited to *Relb* promoter within 30 min of TNF stimulation and then gradually removed (Figure 4a and Supplementary Figure S4d). While early chromatin recruitment was subtle, RelB robustly occupied the *Relb* promoter in *Nfkb2*^−/−^ MEFs at 8 h post-TNF stimulation, corroborating the heightened late RelB DNA-binding activity observed in these cells.

Rewiring this autoregulatory control in our mathematical model, we could efficiently recapitulate progressive nuclear accumulation of the RelB:p50 dimer in *Nfkb2*^−/−^ MEFs in response to TNF (Figure 4b). Computational simulations further predicted intact late RelB:p50 response in *Rela*^−/−^*Nfkb2*^−/−^ MEFs owing to autoregulatory control. Our EMSA confirmed gradual nuclear accumulation with strong RelB:p50 activity at 8 h post-TNF stimulation in *Rela*^−/−^*Nfkb2*^−/−^ MEFs (Figure 4c) and ChIP analyses revealed recruitment of RelB to *Relb* promoter at this time point (Figure 4d and Supplementary Figure S4e). Finally, despite the absence of RelA, TNF-stimulated RelB mRNA synthesis in *Rela*^−/−^*Nfkb2*^−/−^ MEFs (Figure 4e) that temporally coincided with TNF-induced RelB:p50 DNA-binding activity. Collectively, our study identified a novel positive autoregulatory loop, which perpetuates RelB:p50/NFκB activity during canonical TNF signaling (Figure 4f). Such autoregulatory RelB control generated NFκB DNA-binding activity despite inactivation of RelA. Our MEF-based analyses suggested that this autoregulatory loop is hardwired within the NFκB circuit and is triggered upon p100 depletion. We postulated that prolonged RelB:p50 containing NFκB response to TNF in p100-depleted HMCLs could be a consequence of autoregulatory control.

### RelB:p50 dimer is sufficient for TNF-induced expression of NFκB target genes

**Figure 5:**
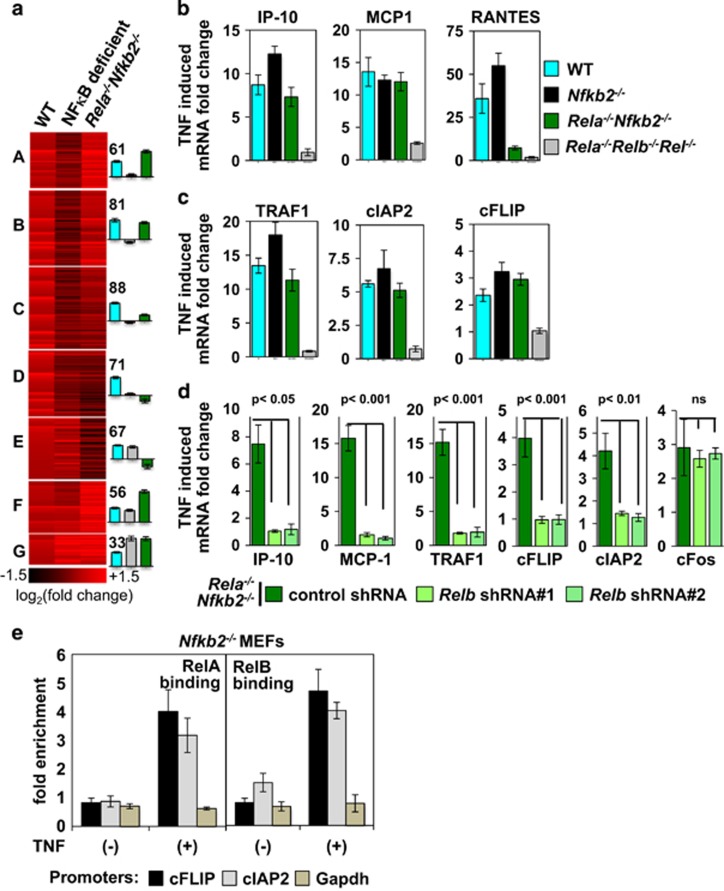
TNF-activated RelB:p50 dimer is sufficient for inducing NFκB target gene expressions. (**a**) Considering genes that are induced at least 1.3-fold at 6 h post-TNF treatment in replicate WT samples with a detection *P*-value <0.05 and signal-to-noise ratio, measured as a ratio of average fold change to standard deviation, ⩾2, we identified 457 genes. Differential expression of these genes between WT, NF-κB deficient (*Rela*^−/−^*Relb*^−/−^*Rel*^−/−^) and *Rela*^−/−^*Nfkb2*^−/−^ MEFs were analyzed by k-median clustering. For individual NF-κB-dependent clusters, median fold changes in gene expression were calculated for various genotypes. Heat map indicating log_2_ (fold induction) values for mRNAs in each cluster. Right, number of genes represented within a given cluster and their mean of fold induction values were compared for these genotypes. (**b** and **c**) Quantitative reverse transcriptase–PCR comparing TNF-induced expression of mRNAs encoding pro-inflammatory cytokines/chemokines IP-10, MCP-1 and RANTES (**b**), and pro-survival mediators TRAF1, cIAP2 and cFLIP (**c**) in WT, *Nfkb2*^−/−^, *Rela*^−/−^*Nfkb2*^−/−^ and *Rela*^−/−^*Relb*^−/−^*Rel*^−/−^ MEFs. mRNA levels were measured at 8 h post-stimulation; except for IP-10 and cFLIP, those were measured at 3 h. The data represent three biological replicates. (**d**) *Rela*^−/−^*Nfkb2*^−/−^ MEFs were transduced with lentivirus-expressing control shRNA or two independent shRNAs targeting RelB. Subsequently, these cells were examined for TNF-induced expression of pro-inflammatory or pro-survival genes at 8 h post-stimulation. cFOS, whose expression is induced by TNF in an NFκB independent manner, was used as a control. The result represents three biological replicates. (**e**) ChIP analyses, representative of three independent biological replicates, revealing recruitment of RelA (left) and RelB (right) NFκB dimers to cFLIP and cIAP2 promoter upon TNF treatment of *Nfkb2*^−/−^ MEFs. Binding to *Gapdh* promoter served as a negative control.

Using genetically tractable MEF system, we investigated how RelB:p50 modulates TNF-induced expression of NF-κB target genes. To this end, we compared global gene expressions (see Materials and methods) in TNF-treated MEFs of the following genotypes, namely WT fibroblasts, which primarily elicits RelA:p50 activity, *Rela*^−/−^*Nfkb2*^−/−^ cells, which exclusively activate RelB:p50, and control NFκB-deficient *Rela*^−/−^*Relb*^−/−^*Rel*^−/−^ cells, which lack the expression of all three transcription-activating NFκB subunits RelA, RelB and cRel. Applying the k-median algorithm to our genome-scale data, we clustered genes induced in WT MEFs at 6 h post-TNF stimulation into seven subgroups (Figure 5a and Supplementary Table S1). Abrogated expression of ~65% of genes belonging to four clusters (cluster A–D) in *Rela*^−/−^*Relb*^−/−^*Rel*^−/−^ cells reiterated a dominant role of NFκB in TNF-induced gene expression. Remarkably, TNF-stimulated gene expression was qualitatively intact in *Rela*^−/−^*Nfkb2*^−/−^ MEFs in three out of four NFκB-dependent clusters. Our quantitative reverse transcriptase–PCR analyses revealed that TNF induces expression of mRNAs encoding pro-inflammatory mediators, such as IP-10, MCP-1 and RANTES (Figure 5b), as well as pro-survival factors, including TRAF1, cIAP2 and cFLIP (Figure 5c), in WT MEFs, but not in *Rela*^−/−^*Relb*^−/−^*Rel*^−/−^ MEFs. Inducible expression of these NFκB target genes was mostly preserved, and if anything was subtly enhanced, in *Nfkb2*^−/−^ cells, which stimulates both RelA:p50 and RelB:p50 activity (Figures 5b and c). Intriguingly, TNF stimulation led to equivalent synthesis of these mRNAs, except for RANTES, in *Rela*^−/−^*Nfkb2*^−/−^ MEFs (Figures 5b and c). shRNA-mediated depletion of RelB abrogated TNF-induced expressions of these genes in *Rela*^−/−^*Nfkb2*^−/−^ MEFs (Figure 5d and Supplementary Figure S5a). However, TNF-induced expression of cFOS, whose transcription is regulated in an NFκB independent manner, remained unaltered upon RelB knockdown in *Rela*^−/−^*Nfkb2*^−/−^ MEFs (Figure 5d and Supplementary Figure S5b). Finally, our ChIP analyses revealed that TNF stimulation indeed promotes recruitment of RelB, in addition to RelA, to promoters of genes encoding cFLIP and cIAP2 in *Nfkb2*^−/−^ MEFs (Figure 5e). Therefore, our gene expression study confirmed that RelB:p50 dimer activated by TNF in p100-deficient cells is capable of inducing expression of a wide spectrum of NFκB target genes, thereby largely circumventing the requirement of RelA. Furthermore, these results extended the possibility that TNF-induced RelB:p50 activity provides for the pro-survival NFκB transcription function in p100-depleted myeloma cells.

### TNF induced RelB:p50/NFκB dimer suppresses apoptosis independent of RelA

**Figure 6:**
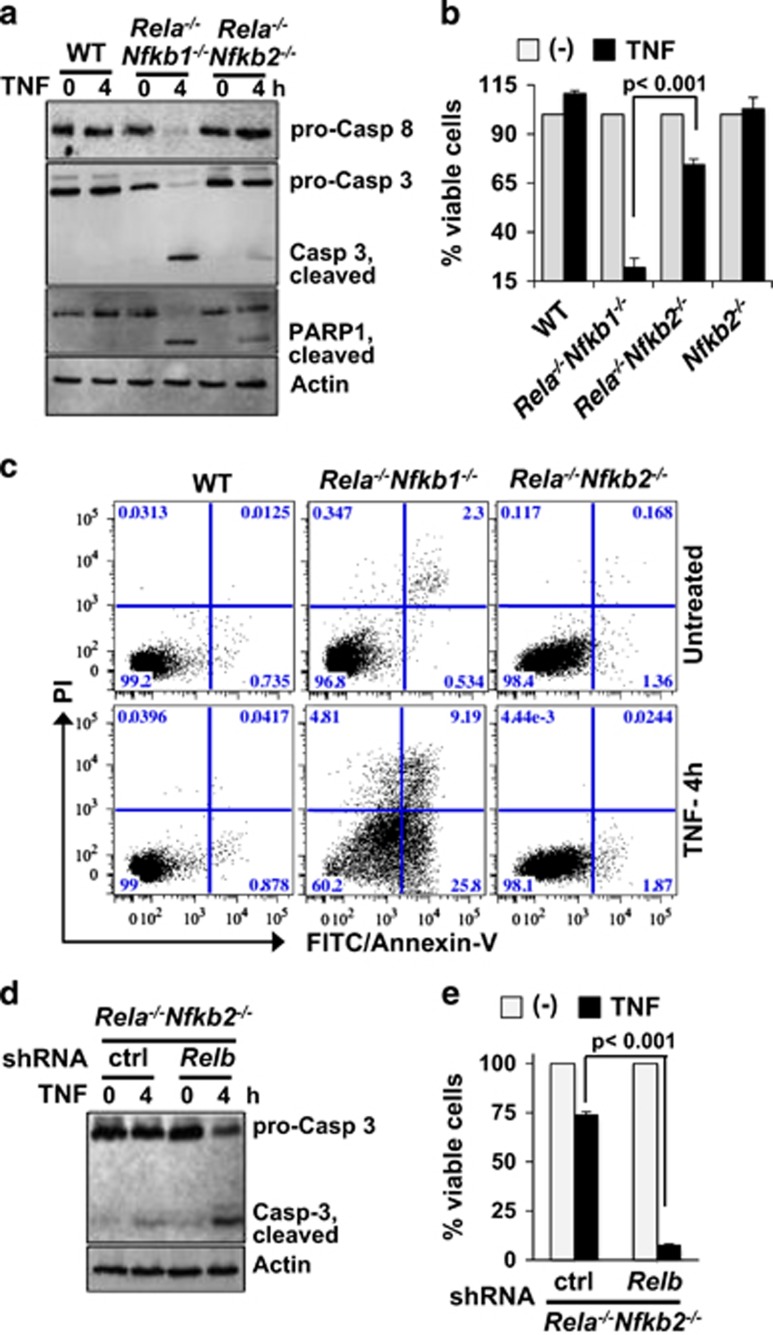
RelB:p50/NFκB dimer protects *Rela*^−/−^*Nfkb2*^−/−^ MEFs from apoptotic death. (**a**) Immunoblot, representative of two experiments, showing differential cleavage of pro-Caspase-8, pro-Caspases 3 and PARP1 in WT, *Rela*^−/−^*Nfkb1*^−/−^ or *Rela*^−/−^*Nfkb2*^−/−^ MEFs upon TNF treatment. Immunoblot of actin served as a loading control. (**b**) MEFs with indicated genotypes were treated with 1 ng/ml of TNF, viable cells were counted at 18 h post-TNF treatment using crystal violet staining and presented relative to untreated cells subsequent to correcting for basal apoptosis. The plot represents average of three independent biological replicates±s.e.m. (**c**) WT and knockout MEFs, left untreated or treated with TNF for 4 h, were examined in fluorescence-activated cell sorting. In a dot plot, representative of three independent experiments, population of cells undergoing apoptosis was scored. (**d** and **e**) Effects of the knockdown of RelB expression using shRNA#2 in *Rela*^−/−^*Nfkb2*^−/−^ MEFs on TNF induced cleavage of pro-Caspases (**d**) as well as cell viability (**e**) were assessed in immunoblot assay and crystal violet staining, respectively. The cell death data are an average of five experimental repeats.

TNF-induced pro-survival factors, such as TRAF1 and cFLIP, counteracts caspase activation in preventing apoptotic cell death.^34^ Sensitivity of *Rela*^−/−^ MEFs to TNF-induced apoptosis underscored the importance of RelA/NFκB-driven pro-survival gene expressions in the cell-death pathway.^35^ Given capacity of the RelB:p50 dimer in stimulating expression of these pro-survival factors, we asked if alternate RelB/NFκB activity suppresses apoptosis in *Rela*^−/−^*Nfkb2*^−/−^ MEFs. As a control, we utilized *Rela*^−/−^*Nfkb1*^−/−^ cells, which lack RelA as well as p50, thus resulting in the deficiency of both RelA:p50 and RelB:p50 dimers (Supplementary Figure S6a). Our immunoblot analyses revealed augmented processing of pro-Caspase-8 and pro-Caspase-3 as well as cleavage of PARP1 upon TNF treatment of *Rela*^−/−^*Nfkb1*^−/−^ MEFs, but not in WT cells (Figure 6a). TNF stimulation of *Rela*^−/−^*Nfkb2*^−/−^ MEFs led to minor change in abundance of pro-Caspases accompanied by only a subtle increase in cleaved Caspase-3 and PARP1. In cell-death assay, we could demonstrate that *Rela*^−/−^*Nfkb2*^−/−^ MEFs are indeed resistant to TNF-induced death, while *Rela*^−/−^ *Nfkb1*^−/−^ cells are extremely susceptible (Figure 6b). Importantly, *Nfkb2*^−/−^ MEFs were impervious to TNF-induced cell death. Our fluorescence-activated cell sorting analyses validated that early apoptosis detected in *Rela*^−/−^*Nfkb1*^−/−^ MEFs within 4 h of TNF treatment is essentially absent in *Rela*^−/−^*Nfkb2*^−/−^ MEFs as in WT cells (Figure 6c and Supplementary Figure S6b). Ablating RelB expression using shRNA, we confirmed that the RelB:p50 dimer is critical for preventing TNF activation of caspases in *Rela*^−/−^*Nfkb2*^−/−^ MEFs (Figure 6d) and protecting these RelA-null cells from apoptosis (Figure 6e). Thus our investigation offered direct genetic evidence that RelB:p50/NFκB activation by TNF in p100-deficient cells is sufficient, and RelA is not required, to suppress the apoptotic cell death.

### Autoregulatory RelB/NFκB pathway promotes pro-survival gene expressions in multiple myeloma

**Figure 7:**
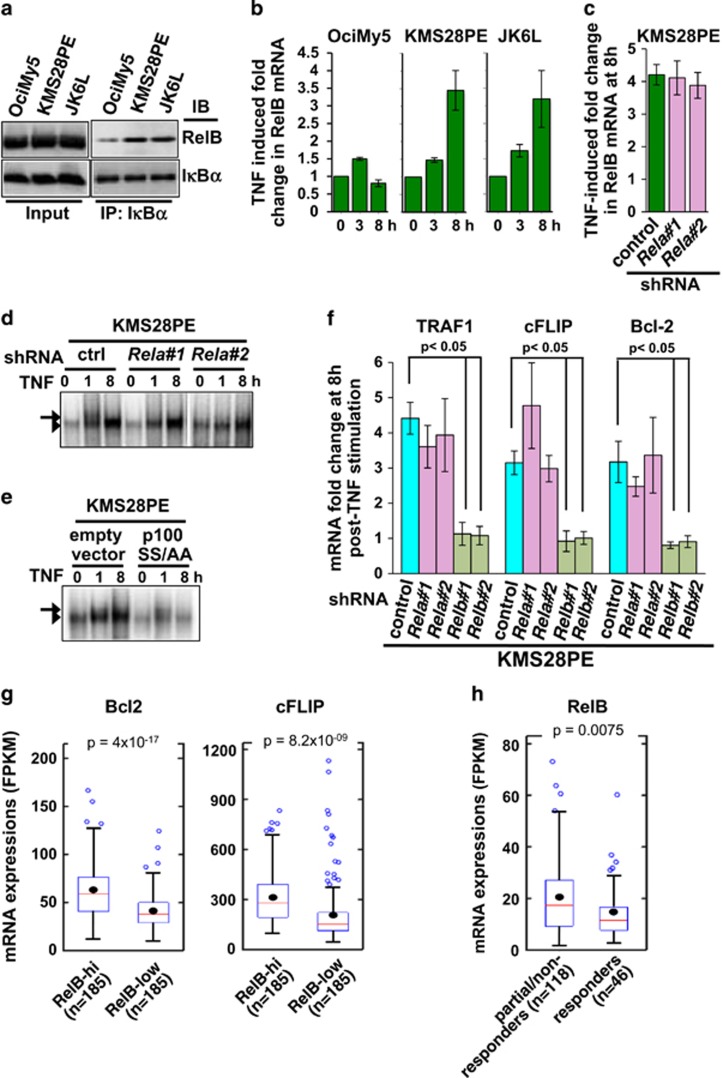
Autoregulatory RelB:p50/NFκB activation in multiple myeloma. (**a**) Immunoblot of IκBα co-immunoprecipitates derived from indicated HMCL extracts and normalized for IκBα content. (**b**) Quantitative reverse transcriptase–PCR revealing TNF induced early (3 h) and late (8 h) expression of RelB mRNA in OciMy5, KMS28PE and JK6L cell lines as an average of three independent biological replicates±s.e.m. (**c** and **d**) TNF-induced expression of RelB mRNA (**c**) and RelB/NF-κB DNA-binding activity (**d**) was measured in KMS28PE cells, which were subjected to shRNA-mediated depletion of RelA using two different shRNAs, as indicated. The data represent three independent biological replicates±s.e.m. (**e**) Electrophoretic mobility shift assay (EMSA) revealing TNF-induced NFκB activation in KMS28PE cells transduced with retrovirus overexpressing a mutant version of p100 (p100_S866A, S870A_), which is unresponsive to non-canonical signals. Cells transduced with empty retrovirus served as a control. (**f**) TNF induced expression of pro-survival genes at 8 h post-stimulation in KMS28PE cells transduced with lentiviral particles expressing control shRNA or shRNAs targeting RelA or RelB. The data represent average of three experiments±s.e.m. (**g**) Levels of Bcl2 and cFLIP mRNA in myeloma patients with high or low RelB mRNA expressions. The RNA-seq data for 371 patients were obtained from the Multiple Myeloma Research Foundation database (themmrf.org). The median RelB expression value was used as a cutoff for cataloguing patients into RelB-high or RelB-low groups. Statistical significance between RelB-hi and RelB-low groups was established using Welch's *t*-test. FPKM indicates fragments per kilobase of exon per million fragments. Filled circle and red line represent mean and median values, respectively. (**h**) Levels of RelB mRNA in myeloma patients grouped into responders, those show complete or stringent complete response to first-line therapy, and partial/non-responder, those exhibit progressive or stable disease or only partial response. The response category of patients, determined using criteria developed by International Myeloma Working Group, and corresponding mRNA data were obtained from themmrf.org database. Gene-expression data represent the mRNA status at the day of diagnosis. Statistical significance was established using Welch's *t*-test.

Our mechanistic studies involving various knockout cells illuminated that p100 deficiency provokes autoregulatory RelB:p50/NFκB activity, which can mediate pro-survival TNF functions. We argued that this mechanism accentuates drug resistance in myeloma. To examine this hypothesis, we first compared IκBα-immunoprecipitates derived from HMCLs for the presence of RelB. Corroborating fibroblast studies, our analyses revealed dominant RelB–IκBα interaction in unstimulated KMS28PE and JK6L cells those lack p100, but not in OciMy5 cells (Figure 7a). Furthermore, perpetuating RelB:p50 response to TNF in KMS28PE and JK6L cells (Figure 2a) temporally coincided with gradual accumulation of RelB mRNA (Figure 7b). TNF stimulated RelB mRNA synthesis (Figure 7c) as well as RelB:p50 DNA-binding activity (Figure 7d) independent of RelA in KMS28PE cells suggesting that autoregulatory RelB control prolongs NFκB response in p100-depleted HMCLs. Conversely, overexpression of a mutant version of p100 (p100_S866A,S870A_), which was resistant to NIK-mediated degradation,^19^ in KMS28PE cells led to a drastic reduction in TNF-induced RelB:p50 NFκB activity (Figure 7e and Supplementary Figure S7). Corroborating our observation that RelB:p50 may provide for pro-survival NFκB function, solitary RelB:p50 activation in the absence of RelA was sufficient for TNF-induced expression of NFκB target genes encoding TRAF1, cFLIP and Bcl2 in KMS28PE cells (Figure 7f). Consistently, shRNA-mediated depletion of RelB, which primarily constitutes the NFκB activity in KMS28PE cells at 8 h post-TNF stimulation, entirely abrogated late induction of these pro-survival factors (Figure 7f). Taken together, p100 depletion reinforced pro-survival TNF response in multiple myeloma by prolonging classical NFκB function via the autoregulatory RelB pathway. Functional redundancy with RelA:p50 dimer coupled to distinct dynamic control allowed RelB:p50 to instill a protracted drug-resistant state in HMCLs.

To further validate our mechanistic hypothesis, we examined gene-expression profile and clinical data of myeloma patients available on the Multiple Myeloma Research Foundation database (themmrf.org). Our analyses revealed that Bcl2 and cFLIP mRNA levels were augmented in the patient group with high RelB mRNA expression as compared with those expressing RelB at a low level (Figure 7g). Moreover, RelB mRNA level was significantly elevated in the group of patients exhibiting progressive or stable disease or only partial response upon first-line therapy as compared with the group showing complete or stringent complete responses (Figure 7h). Given persistently elevated RelB mRNA expression was dependent on autoregulatory synthesis, we argue that the direct correlation of RelB level with pro-survival gene expressions and resistance to therapeutic intervention is consistent to a possible role of the autoregulatory RelB/NFκB pathway in disease pathogenesis of multiple myeloma.

## Discussion

**Figure 8:**
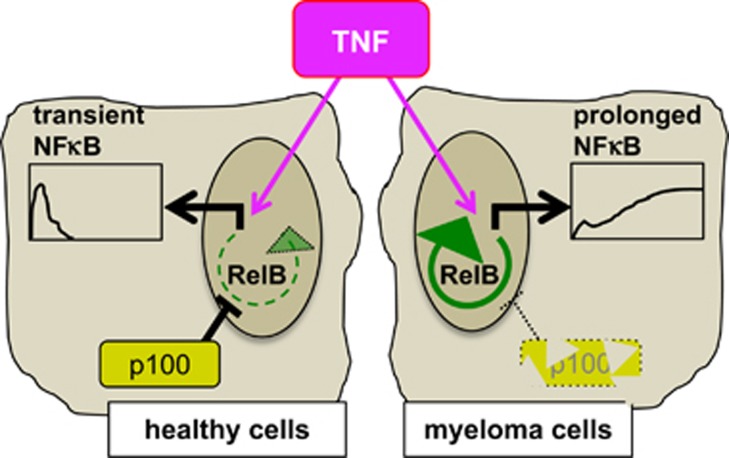
A schematic describing altered dynamic control of NFκB in diseased cells. A model depicting the proposed autoregulatory loop underlying protracted NFκB response to TNF in myeloma. Left, pro-inflammatory cytokines transiently induce NFκB activity, which is composed of RelA:p50 dimer, in healthy cells. Right, genetic aberrations in myeloma deplete p100 to provoke the autoregulatory circuitry in generating protracted RelB:p50/NF-κB response to canonical TNF signals.

Activation of canonical NFκB signaling in myeloma cells by tumor microenvironment derived TNF has been implicated in environmental drug resistance. Recurrent gain-of-function mutations in the non-canonical NFκB module indicated its possible involvement in the resilience of myeloma cells to chemotherapeutic agents. We found that while TNF induces an early NFκB activity, mutational activation of the non-canonical module prolongs TNF-induced pro-survival NFκB response, albeit composed of the RelB:p50 dimer, in exacerbating TRAIL resistance (Figure 8).

Our quantitative analyses indicated that IκBα is a less-preferred interaction partner of RelB, which favors p100 binding (Supplementary Appendix Figures 2b and c). However, depletion of p100, owing to non-canonical mutations, altered the cellular homeostasis with RelB:p50 being sequestered by IκBα and consequently, induced upon TNF stimulation as an early NFκB activity (Figures 2, 3 and 7). Absence of p100 in mutant cells additionally triggered a late-acting RelB:p50/NFκB response, which was contingent upon autoregulatory *Relb* synthesis (Figures 2, 3, 4 and 8). IKK2 was shown to phosphorylate RelB at 6 h post-TNF stimulation that further weakened RelB–IκBα interaction.^36^ It has been suggested that ERK1, which is activated by TNF, also phosphorylates RelB in promoting its nuclear translocation.^26^ Implementing the delayed phosphorylation mechanism in our computational model explained that late-acting RelB:p50 dimer escaped this debilitated IκBα feedback to uninterruptedly synthesize RelB mRNA in cancerous cells.

In a pioneering study, Karin's group earlier demonstrated that expression of cJun proto-oncogene is subjected to positive autoregulation.^37^ An autoregulatory loop connecting IRF4 and MYC was shown to perpetuate oncogenic gene expressions in multiple myeloma.^38^ Epigenetic changes associated with colon cancer were shown to trigger hypoxia-inducible factor-1α autoregulation.^39^ An autoregulatory circuit propagated T-cell acute lymphocytic leukemia protein 1 (TAL1) activity in T-cell acute lymphoblastic leukemia.^40^ Except for *Rela*, genes encoding the NFκB subunits *per se* also bear κB sites at their promoters. In this study, we extended evidence for the involvement of autoregulatory NFκB control in neoplastic diseases. Genetic analyses and mathematical modeling studies substantiated that depletion of p100 in myeloma cells triggers this autoregulatory loop to promote perpetuating RelB:p50/NFκB response to TNF. Strikingly, this TNF-induced, persistent RelB:p50 activity was impervious to RelA inhibitions (Figures 4 and 7d). Our mechanistic investigation indicated that early RelB:p50 activation from IκBα-bound complex is critical for triggering this late-acting RelB:p50 response, particularly when RelA is inactivated.

It was shown that malignant plasma cells possess a low level of NIK-independent, constitutive RelB/NFκB activity. While this activity was initially implicated in expression of NFκB target genes,^27^ a recent investigation suggested that RelB:p52 suppresses expression of NFκB target pro-apoptotic gene Bim.^26^ Indeed, RelB was shown both to activate and inhibit physiological gene expressions from NFκB target promoters,^31, 41, 42, 43, 44^ and RelA, cRel or RelB containing NFκB heterodimers were shown to overlap in their DNA-binding specificity.^45, 46^ However, the perceived notion that RelA is obligatorily required for expression of *Relb*^47^ restricted genetic studies directly evaluating RelB mediated regulations of NFκB target genes in the absence of RelA. Our analyses provided unequivocal evidence that RelB:p50 dimer not only cooperates with RelA:p50 but transcription-activating function of RelB:p50 circumvents the requirement of RelA in mediating pro-survival TNF response in p100-depleted cells, including myeloma cells harboring non-canonical mutations (Figures 5, 6, 7). Although, we do not rule out that RelB, particularly in association with p52, functions as a transcriptional repressor in the basal state in HMCLs, our investigation indicated that TNF-induced RelB:p50 acts as a potent activator of pro-survival gene expressions.

Mutational activation of the non-canonical NFκB pathway modified TNF signaling to impart drug resistance in myeloma cells independent of the principal NFκB subunit RelA. Interestingly, these mutations did not act through typical p52 NFκB complexes, but depleted p100 to reposition RelB under IκBα control. Of note, myeloma-derived cells with enhanced NIK activity were shown to be sensitive to IKK2 inhibitors.^22^ Our study clarified that NIK depletes p100, while IKK2 induces pro-survival RelB:p50/NFκB response from IκBα-inhibited complex. Moreover, autoregulatory RelB:p50 activation by TNF assisted these cells develop autonomy from RelA/NFκB in producing a protracted drug-refractory state. It is thought that the non-canonical RelB/NFκB pathway transduces signal selectively from cell-differentiating cues, and TNF utilizes the canonical RelA/NFκB pathway. We noted that TNF-induced RelA is insufficient for imparting TRAIL resistance (Figures 1 and 2). Rather autoregulatory RelB control mediated precarious collaboration between widespread cell-intrinsic mutations in the non-canonical NFκB module and dynamical signaling induced by TNF in multiple myeloma. The proposed autoregulatory control is consistent to the previous finding demonstrating heightened RelB mRNA levels in myeloma patients harboring mutations in non-canonical NFκB module.^22^ Our observation that high RelB level is correlated with augmented expression of pro-survival factors and resistance of patients to therapy also indicated possible involvement of autoregulatory RelB control in multiple myeloma (Figure 7). In addition to genetic mutations, BAFF and APRIL also activate the non-canonical NFκB pathway in mature B-cells and myeloma cells.^48^ In this context, future studies ought to investigate the possible role of autoregulatory RelB control in transducing BAFF signal in processes requiring prolonged NFκB activity, such as B-cell differentiation and myeloma cell survival.

Our identification that the non-canonical pathway reinforces canonical TNF response through RelB:p50/NFκB dimer reiterates the importance of system-level understanding of pathway integration for designing therapeutic interventions. In sum, highly networked systems controlling functionally redundant transcription factors are also vulnerable to cancerous mutations, which may reverse the feedback control hardwired in the inflammatory circuit. Our results also indicate that acquisition of mutations those provoke autoregulatory activation of pro-survival transcription factors constitutes an important mechanism in environmental drug resistance.

## Materials and methods

### Cells and reagents

Wild-type or gene-deficient C57BL/6 mice were housed at NII and used in accordance with the IAEC guidelines. MEFs, immortalized using 3T3 protocol, were generated from E13.5 embryos, as described.^32^ HMCLs were a kind gift from Dr Michael Kuehl, NCI. Indicated transgenes were either constitutively expressed from MoMLV LTR promoter from pBabe.puro or inducibly expressed from an engineered promoter containing five tandem kappaB sites from HRS.puro retroviral construct. Retrovirus particle were generated from 293 T cells co-transfected with pCL-Eco.^32^ For gene transduction into human-derived myeloma cells, pseudotyped retrovirus particles were produced using Retro-X Universal Packaging System (Clonetech, Mountain View, CA, USA) from GP2-293 cells co-transfected with pBabe.puro construct and Ampho envelope vector (Clonetech). Following shRNA-expressing lentiviral particles (Sigma Aldrich, St Louis, MO, USA) were used for knockdown in HMCLs: *Rela*#1-TRCN0000353629, *Rela*#2-TRCN000029875, *Relb*#1-TRCN0000280360, *Relb*#2-TRCN0000280361, *Nfkb2*#1-TRCN0000356047 and *Nfkb2*#2-TRCN0000356005, and control-SHC202V. For knockdown studies in fibroblasts, lentiviral constructs expressing control shRNA (#RHS4346) or RelB-shRNAs (RMM#4431-101264262 and RMM#4431-200408572) purchased from GE-Dharmacon (Lafayette, CO, USA) were used for generating lentiviral particles.^49^

### Biochemical analyses

MEFs and HMCLs stimulated using 1  and 10 ng/ml of TNF (Roche, Basel, Switzerland), respectively, were harvested, nuclear, cytoplasmic or whole-cell extracts were analyzed by EMSA, supershift analysis, immunoblotting or subjected to immunoprecipitation.^50^ Antibody against p50 (BB-AB0080) was from BioBharati Life Sciences (Kolkata, India). Antibodies against Caspase-8 (#4790), Caspase-3 (#9665) and PARP1 (#9542) were from CST (Danvers, MA, USA).

### Gene-expression analyses

Total RNA was isolated from cells stimulated with 10 ng/ml of TNF and quantitative reverse transcriptase–PCR was conducted, as described,^50^ using cDNA synthesis kit from BioBharati. For microarray analysis, labeling, hybridization to Illumina MouseRef-8 v2.0 Expression BeadChip, data processing and quantile normalization were performed by Sandor Pvt Ltd (Hyderabad, India). Differential expression of genes between different genotypes were analyzed by k-median clustering implemented in the MEV program.^51^ The MIAME version of the microarray data is available on NCBI-GEO (accession GSE68615). For ChIP, 3 × 10^6^ MEFs stimulated with 10 ng/ml of TNF were fixed with 2% formaldehyde, nuclei were isolated, sonicated and lysates were subjected to immunoprecipitation using indicated antibodies (Santa Cruz Biotechnology, Dallas, TX, USA). Subsequent to reverse cross-linking, genomic DNA fragments were extracted from immunopellet and analyzed by qPCR using specific primers.

### Cell-death studies

TNF-induced (1 ng/ml) cell death in MEFs was quantified at 18 h post-stimulation using crystal violet staining, as described.^52^ TRAIL-induced (100 ng/ml; Adipogen, San Diego, CA, USA) cell death in HMCLs was assessed in trypan blue dye exclusion assay. HMCLs were primed with 10 ng/ml of TNF. Apoptosis was scored at 4 h post-stimulation using annexin-V/FITC and propidium iodide (BD Biosciences) staining. Flow cytometry was performed using VERSE (BD Biosciences, Franklin Lakes, NJ, USA) at NII Central facility with the help from A Das, NII, and data were analyzed using FlowJo.

### Computational modeling and code availability

The NF-κB Systems Model *v*2.0 was developed based on previously published models,^31, 50^ refined using our experimental data and was simulated in Matlab. A detailed description including parameter values has been provided in Supplementary Appendix. Matlab codes are available upon request.

### Statistical analysis

Error bars are shown as s.e.m. of 3–5 biological replicates, which was determined according to the standard practices for cell-culture-based analyses. Exact numbers of replicates for each experiment have been indicated in the legends of the corresponding figures. Statistical significance between groups was calculated using two-tailed Student's *t*-test and the *P*-values have been mentioned in the respective figures. For analyses of patients data obtained from Multiple Myeloma Research Foundation, Welch's *t*-test was used.
