# Supplementary material for: Non-canonical NFκB mutations reinforce pro-survival TNF response in multiple myeloma through an autoregulatory RelB:p50 NFκB pathway
Source: Oncogene. 2016 Sep 19;36(10):1417–29. doi: 10.1038/onc.2016.309 (PMC5346295; doi:10.1038/onc.2016.309)
Supplement: Supplementary Information [file onc2016309x1.pdf]

Non-canonical NFκB mutations reinforce pro-survival TNF response in multiple myeloma through an autoregulatory RelB:p50 NFκB pathway

Payel Roy[1], Tapas Mukherjee[1], Budhaditya Chatterjee[1,2], Bharath Vijayaragavan[1], Balaji Banoth[1], Soumen Basak[1, 3]

[1]Systems Immunology Laboratory

National Institute of Immunology

Aruna Asaf Ali Marg

New Delhi-110067

India

[2] Current Address: Kusuma School of Biological Sciences, IIT-Delhi

Hauz Khas, New Delhi, India

[3] Current Address: The Weizmann Institute of Science, Israel

[3] Correspondence should be addressed to S.B. (e-mail: sobasak@nii.ac.in)

tel: (91) (11) 26703853; fax: (91) (11) 26742626

<u>**Supplementary Figures and Table**</u>

I. Figure legends for Supplementary Figures S1-S7 related to Figures in the main text.

II. Supplementary Table S1 related to gene expression studies described in Figure 5.

III. Supplementary Figure S1-S7

I. Figure legends for Supplementary Figures S1-7

Figure S1: TRAIL induced cell-death in TNF-primed HMCLs

- **a.** A time-course analyses demonstrating TRAIL induced cell-death in OciMy5, KMS28PE, JK6L, KMS20 and OciMy1 HMCLs. Cell-death was assessed in trypan blue dye exclusion assay. Basal cell-death in untreated cells has also been presented. The results represent average of five biological replicates ± SEM.
- **b.** KMS28PE and JK6L cells, untreated or TNF stimulated for 8h were stained using FITC tagged Annexin-V antibody and PI and subjected to FACS. Dot plot representing, three independent experiments, captures a lack of apoptotic death upon solitary TNF treatment.

Figure S2: Investigating perturbed NFκB system HMCLs

- **a.** EMSA revealing nuclear NFκB activity induced upon treatment of JK6L cell-line with PMA/Ionomycin. Arrow and arrowhead represent RelA and RelB containing DNA binding activities.
- **b.** Quantitative RT-PCR comparing basal NFκB2 mRNA levels in OciMy1, OciMy5, KMS20, KMS28PE and JK6L HMCLs. Data represents three biological replicates.
- **c.** and **e.** Immunoblot revealing RelA **(c)** or RelB **(e)** levels in whole cell extracts derived from KMS28PE subjected to lentivirus mediated knockdown of RelA or RelB expressions, using shRNAs *Rela*#1 (Cat No. TRCN0000353629 from Sigma Aldrich) and *Rela*#2 (Cat No. TRCN000029875) or shRNAs *Relb*#1 (Cat No. TRCN0000280360) and *Relb*#2 (Cat No. TRCN0000280361), respectively. Control shRNA was also from Sigma Aldrich (Cat No. SHC202V).

d. Quantitative RT-PCR comparing NFκB2 mRNA levels in KMS28PE cells transduced with lentivirus expressing a control shRNA or shRNAs targeted against NFκB2 (*Nfkb2*#1, Cat No. TRCN0000356047 and *Nfkb2*#2 Cat No. TRCN0000356005). Results represent average of three biological replicates ± SEM. Given low level of expression of p52/p100 in these myeloma cells, mRNA analyses were conduced to evaluate efficiency of knockdown.

Figure S3: IκBα and RelA dependent regulation of RelB NFκB activity

a. Immunoblot of RelB co-immunoprecipitates derived from whole cell extracts demonstrating RelB binding to IκBα and IκBε, but not IκBβ, in *Nfkb2*$^{-/-}$ MEFs.

b. Composition of NFκB DNA binding activity induced in *Nfkb2*$^{-/-}$ MEFs upon 30min of TNF stimulation was examined by supershift assay. Combining antibodies against indicated RelB subunits with αRelA antibody, we identified nuclear activation of RelB:p50 containing NFκB DNA binding complex.

c. Quantitative RT-PCR comparing TNF induced expression of RelB mRNA at 3h post-stimulation in WT and *Rela*$^{-/-}$*Nfkb1*$^{-/-}$ MEFs. Results represent average of four biological replicates ± SEM.

d. TNF induction of RelB mRNA stably expressed from either a constitutive or an NFκB inducible promoter from retroviral transgenes in *Relb*$^{-/-}$*Nfkb2*$^{-/-}$ MEFs at 3h post-stimulation. The results in (**c**) and (**d**) represent average of three experiments ± SEM.

e. A Co-immunoprecipitates derived from TNF stimulated WT extracts using an antibody raised against the C-terminal region of p100 was immunoblotted for RelB.

Figure S4: An autoregulatory loop underlying RelA independent RelB:p50 activation

a. Aligning *Relb* promoter DNA sequences derived from human and mouse genomes, two conserved κB sites were identified, as described earlier by Bren *et. al.*, 2001. '*' denote the transcription start sites; proximal as well as distal κB motifs were enclosed in boxes.

b. Oligo competition assay comparing endogenous RelA:p50 and RelB:p50 dimers, simultaneously activated upon 4h of TNF treatment in *Nfkb2*$^{-/-}$ MEFs, for their ability to bind to κB sites derived from *Relb* promoter. Challenging with indicated unlabeled oligos, those harbor sequences encompassing either proximal or distal κB sites present in *Relb* promoter, similarly affected both the dimers for their ability to bind to radiolabelled consensus κB DNA probe in EMSA. The data represents three independent experiments.

c. Specificity of oligo competition assay was ensured using competitor oligos containing a consensus κB site or its mutant variant.

d. Experimental data corresponding to ChIP analyses presented in Figure 4a has been plotted relative to input signals. The data, representative of three biological repeats, demonstrates recruitment of RelA (left) and RelB (right) NFκB dimers to *Relb* promoter in a TNF time course in *Nfkb2*$^{-/-}$ MEFs.

e. Experimental data corresponding to ChIP analyses presented in Figure 4d has been plotted relative to input signals. The data, representative of three independent expriments, demonstrates recruitment of RelB dimers to *Relb* promoter at 8h post-TNF stimulation in *Rela*$^{-/-}$*Nfkb2*$^{-/-}$ MEFs.

Figure S5: shRNA mediated knockdown of RelB expression

a. Immunoblot for RelB of lysate prepared from *Rela*$^{-/-}$*Nfkb2*$^{-/-}$ MEFs transduced with lentivirus expressing a control shRNA (#RHS4346 from GE-Dharmacon) or shRNAs targeting RelB (RMM#4431-101264262 and RMM#4431-200408572 from GE-

Dharmacon). Five fold concentrated cell extracts were used for critically evaluating the efficiency of knockdown.

b. Quantitative RT-PCR demonstrating that TNF induces expression of cFOS in an NFκB independent manner. Expression of cFOS mRNA was similarly induced by TNF in WT, *Rela*$^{-/-}$*Relb*$^{-/-}$*Rel*$^{-/-}$ (NFκB-deficient) and *Rela*$^{-/-}$*Nfkb2*$^{-/-}$ MEFs. The results represent an average of four experiments ± SEM.

Figure S6: Functional redundancy between RelA and RelB NFκB dimers

a. EMSA revealing a lack of TNF induced NFκB activity in *Rela*$^{-/-}$*Nfkb1*$^{-/-}$ MEFs.

b. MEFs of the indicated genotypes were left untreated or treated with TNF for 4h and subsequently stained using FITC-Annexin-V antibody and PI. Apoptotic (Annexin-V^{+} and Annexin-V^{+}/PI^{+}) cell death was quantified using FACS. Results reflect mean of three biological replicates ± SEM.

Figure S7: Reintroduction of a signal-unresponsive mutant of p100 into KMS28PE cells

Immunoblot revealing level of p100 in KMS28PE cells transduced with retrovirus expressing a mutant of p100 ($p100_{S866A,\ S870A}$), which is unresponsive to non-canonical NIK signals. Cells transduced with empty retrovirus served as a control.

II. Supplementary Table S1 related to gene expression studies described in Figure 5.

Table S1: TNF induced genes grouped into different clusters based on their differential expressions in WT, NFκB-deficient (*Rela*$^{-/-}$*Relb*$^{-/-}$*cRel*$^{-/-}$) and *Rela*$^{-/-}$*Nfkb2*$^{-/-}$ cells

Cluster A	Cluster B	Cluster C	Cluster D	Cluster E	Cluster F	Cluster G
Dcn	Prrx1	Man2a2	Flrt3	Atg2a	Daam1	Rrp1b
Gadd45b	Hsd17b14	Ralb	Cish	Ptk9	Cd93	Slco3a1
Slc25a37	Igf2bp1	Rufy3	9130422G 05Rik	H2-Q7	Letm1w	Mdn1
Ddx58	1500003O03 Rik	Cd274	Pigc	Slc25a30	Dusp22	Cyr61
Cdkn2b	Ninj1	LOC1000 46406	Garnl4	Ptpn12	Polm	Tbc1d1
Hnrpdl	Maea	Parp3	Ing5	Gigyf1	Fjx1	Lrrc8a
1110018G 07Rik	Olfr802	Hoxb3	Pcnx	Dnajb6	Ass1	Ccrn4l
Atp7a	Tmed8	Slit2	Cebpb	Abcb1b	Snx11	Oaf
Tjp2	Ext1	LOC1000 45950	Chd8	Srebf2	Gclm	LOC6696 60
Mgat4b	Eif4ebp2	Cdkn2a	Dusp8	Hist1h1b	Coq10b	Plk3
Rsf1	Slc35b1	Clcn3	Bid	Sqstm1	Klf5	Plaur
Birc2	Rab6b	9030612M	1200002N	Phf21a	2610103J23	Dusp7

		13Rik	14Rik		Rik	
Pnpla2	Arid3a	Kifc3	Gdpd1	Ankrd13c	Psmd8	Nup54
Rab12	LOC100044177	Papss2	A330021E22Rik	Mdh2	Mib1	Ercc8
LOC100046393	Qrich1	Stard3	Prss23	Pscd3	Cdc25a	Cx3cl1
Dclre1c	Phf19	Snx13	Gpld1	Olfr965	Rapgef3	Sgtb
Gats	Rab32	Gkap1	Cnot4	Cables2	Camkk2	Senp5
Nfkb1	Taf15	Antxr1	Foxp1	Ssx2ip	Rbm38	Fkhl18
Fam134b	Itgav	Ppfibp1	Synm	Itgb1	Orai1	LOC100045780
R3hdm1	Ccdc109a	Psmd10	Arhgef3	Mt2	Bcl10	Denr
Dlgap4	Lpin2	Slc38a2	Adam17	Ehd1	Chuk	Aoc2
Hpx	Cldn7	Prkcd	Rnmt	Slc16a9	Tbl3	Slc25a33
Mtss1	Pard3	Mars	Ier3	2810439F02Rik	Fubp1	Bop1
Golga7	Rapgef6	Rab1	Napb	Nfkbib	Dync1li1	2310061F22Rik
4930570C03Rik	Gabra3	Enpp4	Ppap2b	Atp2b1	Smox	Lass6
P4ha2	Apoc2	Psmb10	Apob48r	Eif2c2	Cdc34	Kpna1
Dnajc7	Foxp4	Pank3	Hp	Sdc4	Tmem199	Pdcd11
Ddb1	Ccnd2	1190003J1	Dennd3	Cd34	Ccl2	Rasa2

		5Rik				
Nadk	Epb4.1l5	Stat5a	Gna13	Dusp16	Adora2b	Inhba
Fktn	Rnpc2	Rffl	Herc2	LOC100048726	Hipk2	Mllt11
Lmtk2	Gfod1	Cd82	Sp6	LOC100048020	Dot1l	Micall2
Clip1	Col5a3	Btbd3	Afap1	Gch1	Gpn2	Mthfd2
BC031575	9030625A04Rik	Cxcl1	Rnd3	Axud1	2510010F15Rik	Prei3
Sod2	Mapkapk2	Jmy	Cd14	LOC677144	A030007L17Rik	
Tapbp	Tank	Rela	Slc29a1	Dock4	2610028A01Rik	
Mafg	Grit	1110008P14Rik	Fam20b	EG433923	Tbrg4	
Igsf3	Dbn1	0610007P08Rik	Sec22c	Nomo1	Stk4	
Tbl1x	Cacna2d1	EG665378	Fndc3b	Lpcat4	Rbm13	
Stard4	Sec23ip	Gtpbp5	Egr1	Cfl2	Msn	
Bst2	Fstl1	Nek8	Sh3pxd2b	Styx	Rab6	
BC038156	Pmpca	Rnf114	Rpia	Rasa1	Commd2	
Nfe2l1	Ifit3	Il6st	Arrb2	BC004044	Psmd1	
Lphn1	Heca	Plekhg2	Tbc1d20	Myef2	Ddx21	

Pcmtd1	App	Fam178a	Slc27a3	Nid1	Stk40	
Mllt1	Rrbp1	Nfatc3	Irf5	Rtn4ip1	Mak16	
Mcc	Adamts7	4930519N13Rik	Kif5c	Rap1b	Ppp2r5b	
Stat2	Gsdmdc1	LOC639396	D16Ertd472e	Tmem23	Snora65	
Bcl2l2	Map4k2	Atxn1	Arhgef4	LOC100047762	Ppp2r1b	
Nod1	Map3k3	Bbx	Stk38l	Syngr2	Mtdh	
BC021381	Ptges2	Agrn	Entpd7	Edg5	Rrp12	
LOC216443	Hmgn3	Syt11	Lrp6	Cdc42ep3	Ddx19b	
Lsm14b	Pltp	Mapre2	Hus1	St3gal1	Cdc37	
Setd2	Ogt	Fst	Sirt7	LOC100044468	Polr3g	
Chka	Msi2	Nfkb2	Rcor1	Hnrph1	Purb	
Cast	Psme1	Ifi35	Sema4a	Phldb1	Mrpl38	
Ankrd25	Mmp3	Casp7	Il1rap	Stam	Slc25a25	
Gpc1	Rnu6	Pip5k1a	Samd4b	LOC621823		
C3	Elf2	Akna	Lnpep	Snrp70		
Map3k7ip2	Eif2c3	Ank2	Cfp	Baz1a		

Egr3	Tm9sf4	Nup93	Il13ra1	Mapk11		
Srp54	Nfkbia	Bcl2l13	Secisbp2			
Ifngr1	Dcbld2	Asf1a	Pigl			
Gnptab	Gdpd5	Lnx2	Numb			
Lrp11	Sc4mol	Tlr2	Nr6a1			
BC037034	Slc11a2	Rspo3	Rpn1			
Gphn	Glrx	Csnk1g1	Slc9a3r1			
Polr3a	LOC100047963	Lonrf1				
Map2k2	Ppp1r16a	BC006779				
Iigp2	LOC100039728	Gadd45a				
Ccl7	Furin	Alas1				
Arid5b	Arhgap22	N4bp2				
Sema4b	Ap3m1					
Nab1	Olfr952					
Lhfpl2	LOC100047856					
5133401N09Rik	Tfrc					
Tkt	Myo10					
Stat3	Csf1					

Aars	Plscr1					
Rhbdl2	Gsk3b					
BC017647	Lime1					
Htt	Mvp					
B230380D07Rik						
Mobkl2c						
Rif1						
Irf1						
1200016D23Rik						
Ikbke						
Ets2						

III. Supplementary Figures

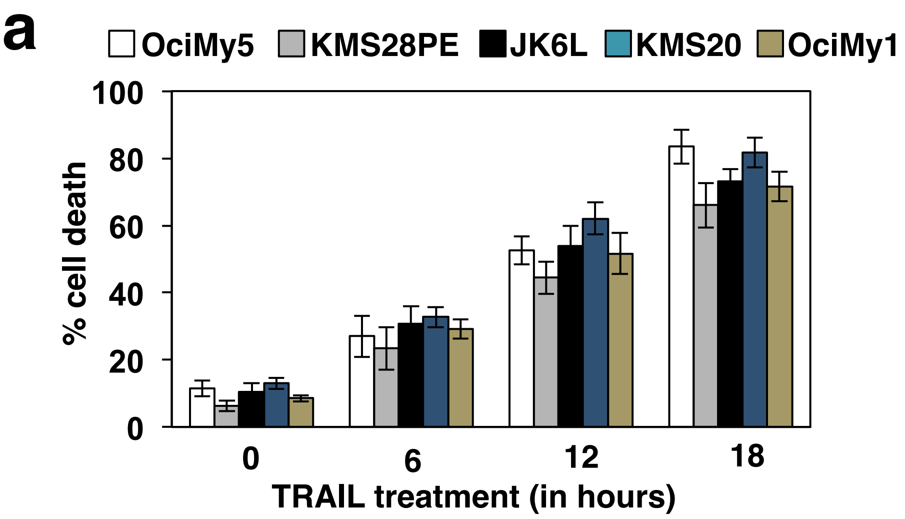

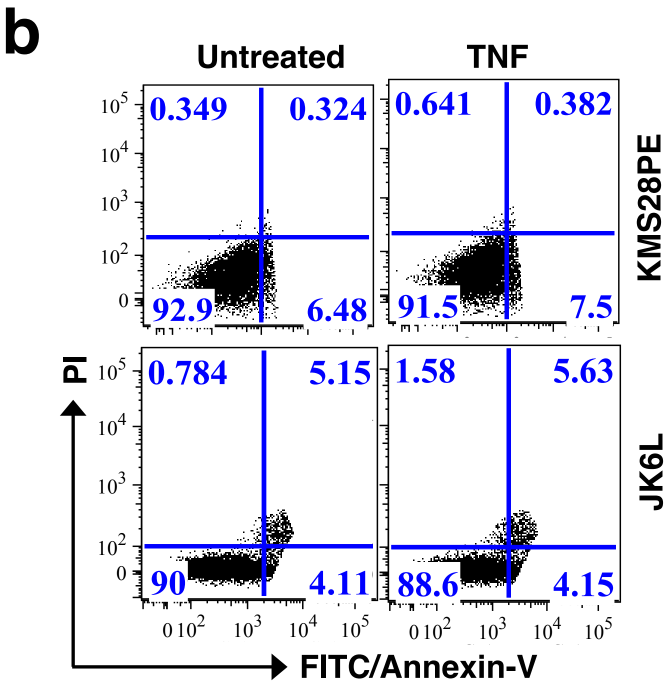

Figure S1

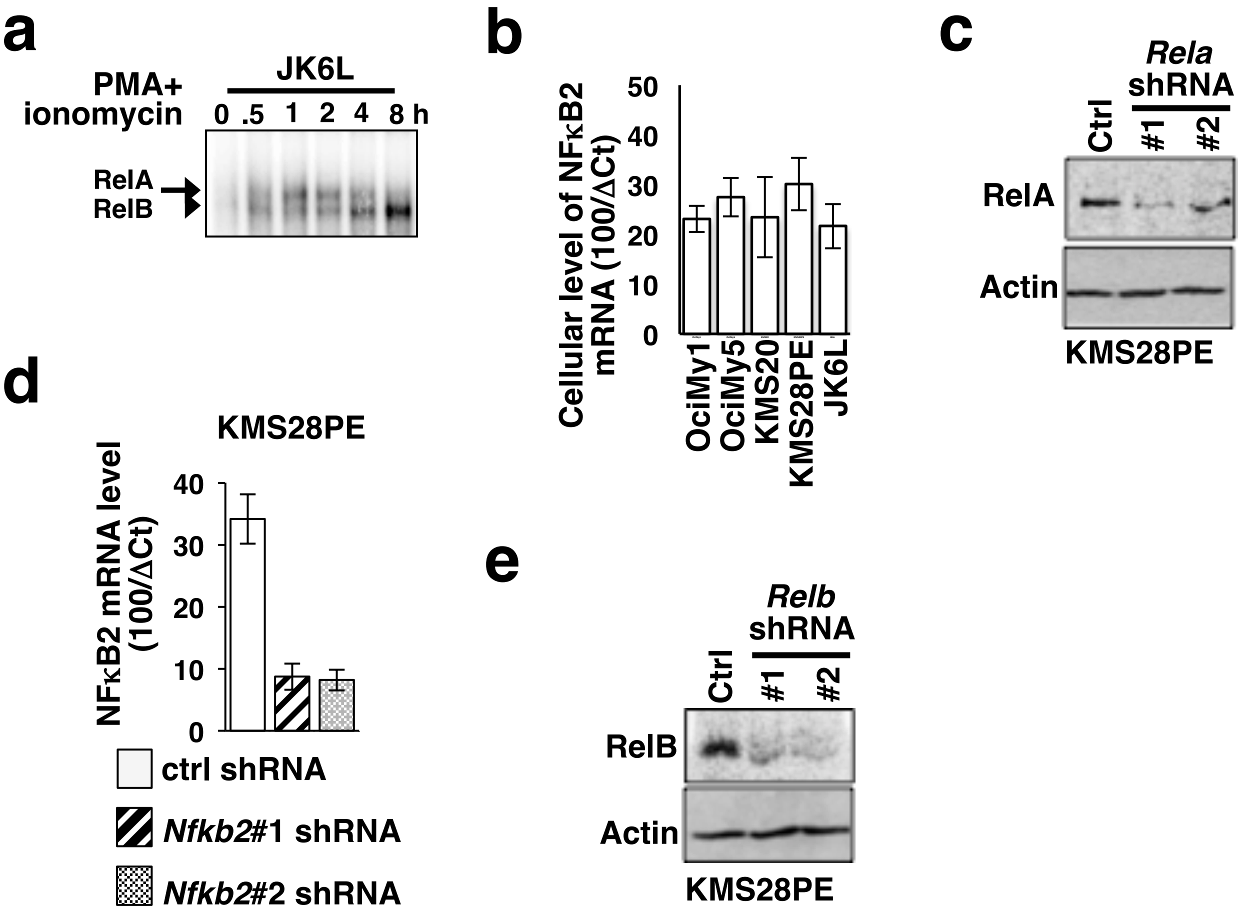
a
PMA+
ionomycin
JK6L
0 .5 1 2 4 8 h
RelA
RelB
b
Cellular level of NFκB2 mRNA (100/ΔCt)
50
40
30
20
10
0
OciMy1
OciMy5
KMS20
KMS28PE
JK6L
c
Ctrl
Rela shRNA
#1
#2
RelA
Actin
KMS28PE
d
KMS28PE
NFκB2 mRNA level (100/ΔCt)
40
30
20
10
0
ctrl shRNA
Nfkb2#1 shRNA
Nfkb2#2 shRNA
e
Ctrl
Relb shRNA
#1
#2
RelB
Actin
KMS28PE

Figure S2

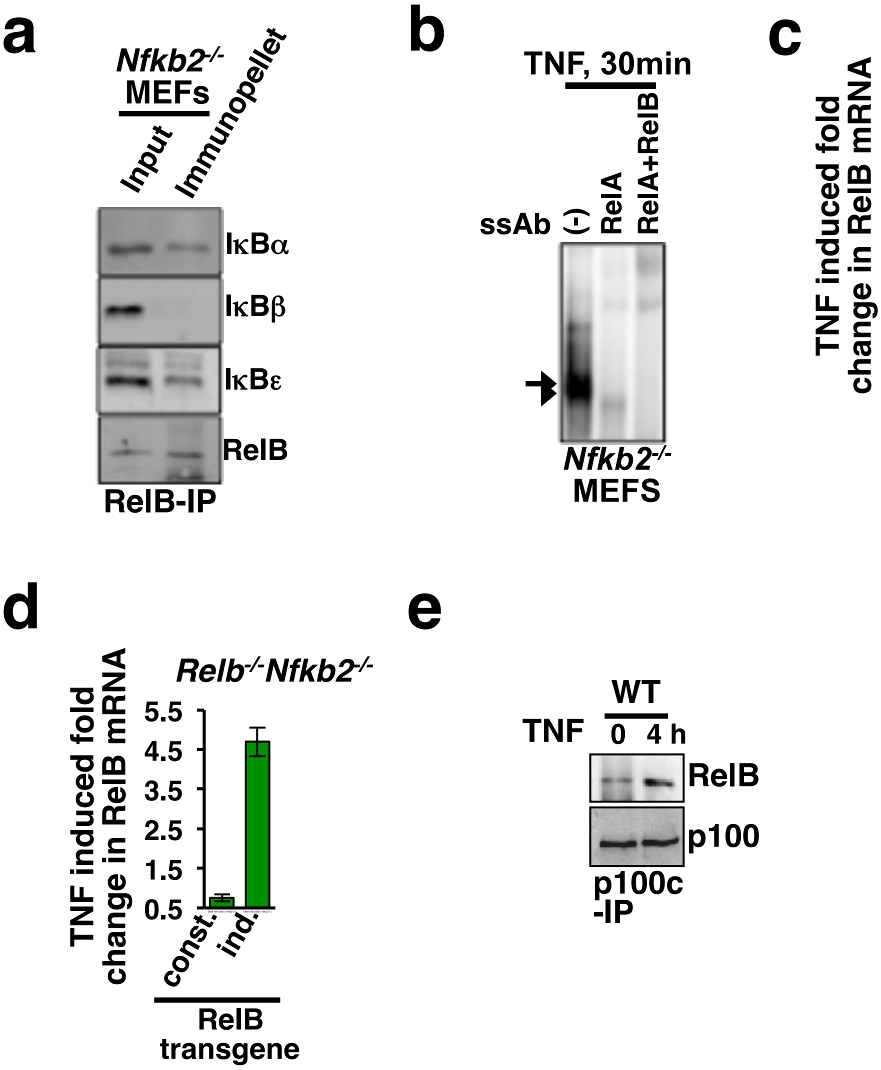
a
Nfkb2-/- MEFs
Input
Immunopellet
IκBα
IκBβ
IκBε
RelB
RelB-IP
b
TNF, 30min
ssAb
(-)
RelA
RelA+RelB
Nfkb2-/- MEFS
c
TNF induced fold change in RelB mRNA
5.5
4.5
3.5
2.5
1.5
0.5
WT
Rela-/- Nfkb1-/-
d
Relb-/-Nfkb2-/-
TNF induced fold change in RelB mRNA
5.5
4.5
3.5
2.5
1.5
0.5
const.
ind.
RelB transgene
e
WT
TNF
0
4 h
RelB
p100
p100c -IP

Figure S3

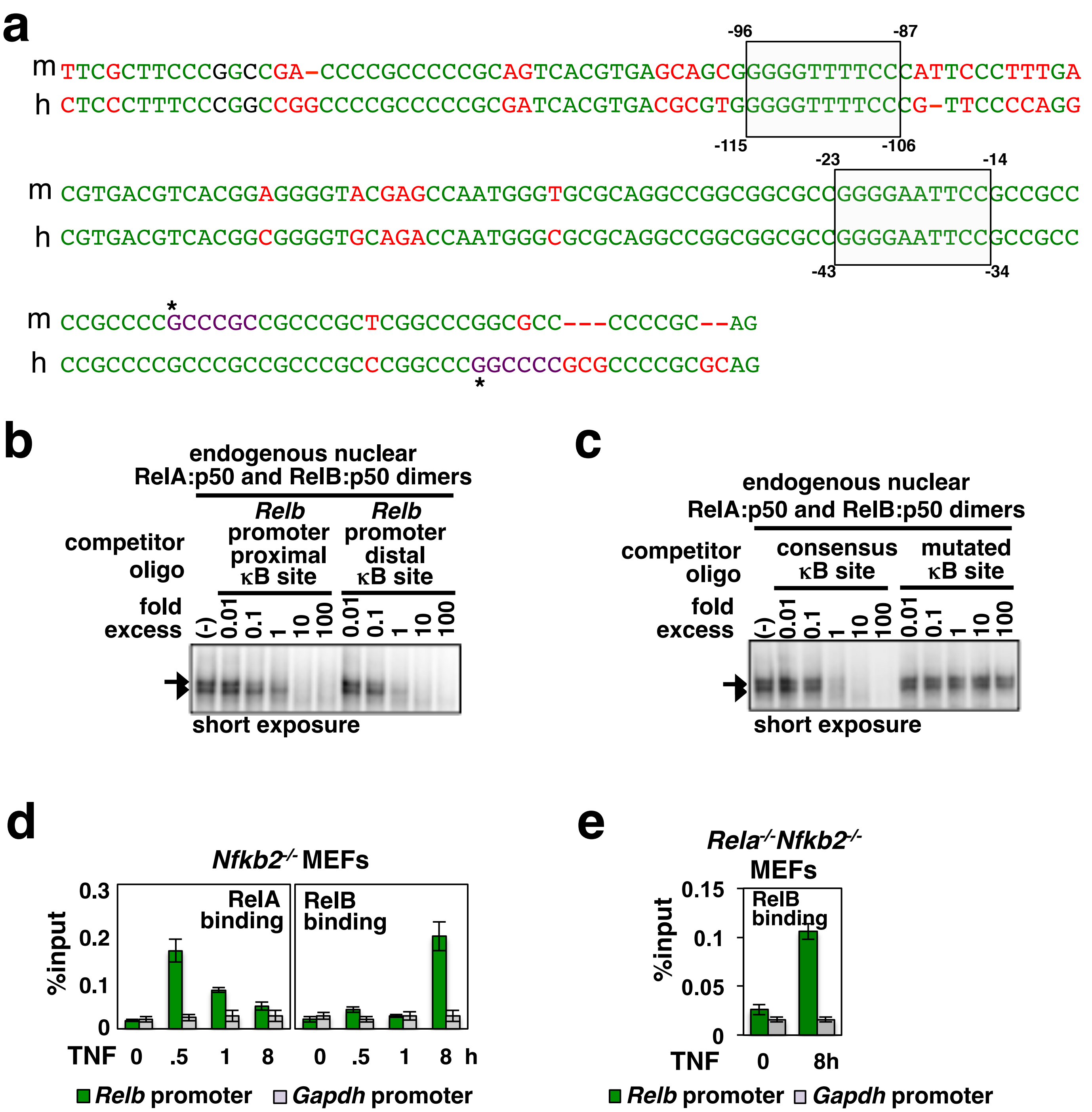
a
-96
-87
m TTCGCTTCCCGGCCGA-CCCCGCCCCCGCAGTCACGTGAGCAGCGGGGGTTTTCCCATTCCCTTTGA
h CTCCCTTTCCCGGCCGGCCCCGCCCCCGCGATCACGTGACGCGTGGGGGTTTTCCCG-TTCCCCAGG
-115
-106
-23
-14
m CGTGACGTCACGGAGGGGTACGAGCCAATGGGTGCGCAGGCCGGCGGCGCCGGGGAATTCCGCCGCC
h CGTGACGTCACGGCGGGGTGCAGACCAATGGGCGCGCAGGCCGGCGGCGCCGGGGAATTCCGCCGCC
-43
-34
*
m CCGCCCCGCCCGCCGCCCGCTCGGCCCGGCGCC---CCCCGC--AG
h CCGCCCCGCCCGCCGCCCGCCCGGCCCGGCCCCGCGCCCCGCGCAG
*
b
endogenous nuclear RelA:p50 and RelB:p50 dimers
competitor oligo
Relb promoter proximal κB site
Relb promoter distal κB site
fold excess
(-) 0.01 0.1 1 10 100 0.01 0.1 1 10 100
short exposure
c
endogenous nuclear RelA:p50 and RelB:p50 dimers
competitor oligo
consensus κB site
mutated κB site
fold excess
(-) 0.01 0.1 1 10 100 0.01 0.1 1 10 100
short exposure
d
Nfkb2-/- MEFs
%input
0.3
0.2
0.1
0
RelA binding
RelB binding
TNF 0 .5 1 8 0 .5 1 8 h
Relb promoter
Gapdh promoter
e
Rela-/-Nfkb2-/- MEFs
%input
0.15
0.1
0.05
0
RelB binding
TNF 0 8h
Relb promoter
Gapdh promoter

Figure S4

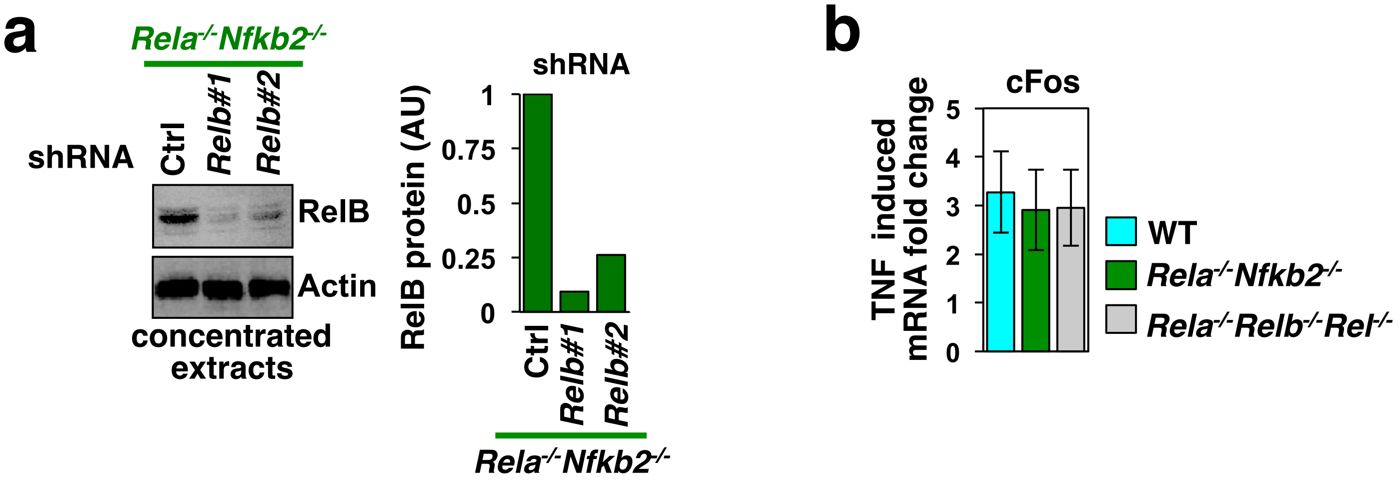
a
Rela-/-Nfkb2-/-
shRNA
Ctrl
Relb#1
Relb#2
RelB
Actin
concentrated extracts
shRNA
RelB protein (AU)
1
0.75
0.5
0.25
0
Ctrl
Relb#1
Relb#2
Rela-/-Nfkb2-/-
b
cFos
TNF induced mRNA fold change
5
4
3
2
1
0
WT
Rela-/-Nfkb2-/-
Rela-/-Relb-/-Rel-/-

Figure S5

a

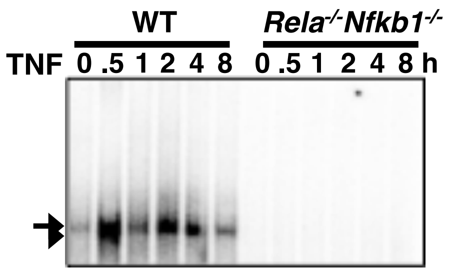
WT
Rela-/-Nfkb1-/-
TNF 0 .5 1 2 4 8 0 .5 1 2 4 8 h

b

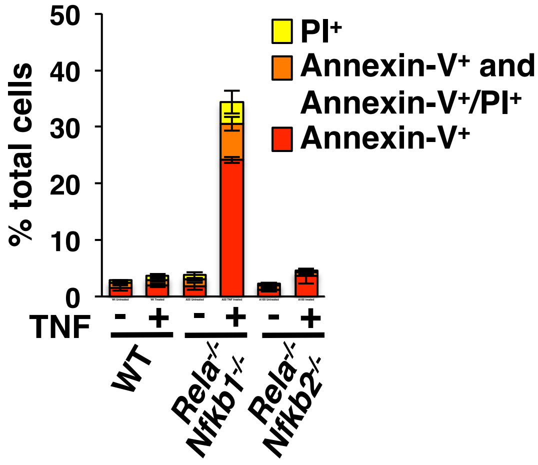
50
40
30
20
10
0
% total cells
PI+
Annexin-V+ and
Annexin-V+/PI+
Annexin-V+
TNF
- + - + - +
WT
Rela-/-
Nfkb1-/-
Rela-/-
Nfkb2-/-

Figure S6

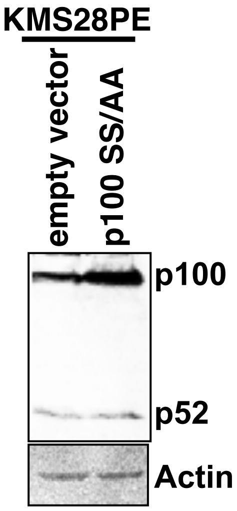

Figure S7
